# Patterns and clinical outcomes of antidepressant prescribing in children and adolescents: real-world evidence from a mental health hospital in Türkiye

**DOI:** 10.3389/fpsyt.2026.1947706

**Published:** 2026-10-29

**Authors:** Asiye Arıcı Gürbüz, Hatice Altun

**Affiliations:** 1 Department of Child and Adolescent Psychiatry, Adana Dr. Ekrem Tok Mental Health Hospital, Adana, Türkiye; 2 Department of Child and Adolescent Psychiatry, Faculty of Medicine, Kahramanmaras Sutcu Imam University, Kahramanmaras, Türkiye

**Keywords:** adolescent, antidepressants, child, off-label use, real-world evidence, SSRI, Türkiye

## Abstract

**Background:**

Antidepressant use in child and adolescent psychiatry has increased diagnoses, antidepressant data from Türkiye remain scarce. This study aimed to describe antidepressant prescribing patterns and to explore clinical and demographic factors associated with clinical improvement, off-label use and adverse effects in a child and adolescent psychiatry outpatient population.

**Methods:**

This single-centre retrospective study reviewed the records of 650 patients aged 4–17 years prescribed antidepressants at a public mental health hospital between August 2020 and December 2021.Demographic characteristics, psychiatric diagnoses, antidepressant agents and doses, off-label status (FDA paediatric labelling),concomitant psychotropic treatment, adverse effects and Clinical Global Impression (CGI) ratings were extracted. A positive response was defined as a CGI-Improvement (CGI-I) score of 1–2 at the last recorded follow-up. Multivariable logistic regression was used to explore factors associated with CGI-I response, off-label prescribing and adverse effects.

**Results:**

Most patients were female (58.9%), and 43.8% were aged 15–17 years; the mean age was 13.23 ± 3.18 years. At least one selective serotonin reuptake inhibitor (SSRI) was prescribed to 92.5% of patients, mainly fluoxetine (48.2%) and sertraline (34.2%).Anxiety disorders (41.8%),major depressive disorder (MDD; 37.8%) and attention-deficit/hyperactivity disorder (ADHD;28.8%) were the most frequent diagnoses. Prescribing was off-label in 74.8%, and 43.8% received concomitant psychotropic treatment. Adverse effects, mainly sedation and weight gain, occurred in 21.4% (monotherapy 11.8% vs. concomitant treatment 33.7%).A positive CGI-I response was recorded in 84.6%;without a control group, this does not indicate efficacy. Relative to age 4–10 years, age 11–14 years (aOR 0.37,95%CI 0.17–0.83) and 15–17 years (aOR 0.28,95% CI 0.13–0.61), and a prior antidepressant trial (aOR 0.46,95%CI 0.27–0.78),were associated with lower odds of a positive response. Off-label prescribing was more likely in generalised anxiety disorder (aOR 2.41,95%CI 1.35–4.30) and less likely in MDD (aOR 0.31,95% CI 0.21–0.47).Adverse effects were associated with concomitant treatment (aOR 3.32,95% CI 2.14–5.16) and ADHD (aOR 1.77,95%CI 1.17–2.69).

**Conclusions:**

In this single-centre sample, antidepressant prescribing was most common among female patients and older adolescents, dominated by SSRIs, frequently off-label and often combined with other psychotropics. Younger age was associated with greater clinical improvement, and concomitant treatment with adverse effects. These exploratory, non-causal findings highlight the need for prospective, multicentre studies with standardised outcome assessment to inform prescribing guidelines.

## Introduction

The prevalence of psychiatric disorders among children and adolescents has increased markedly in recent years. When left untreated, these conditions can adversely affect academic achievement, family dynamics, social functioning, and overall developmental trajectories. Some of the most common psychiatric diagnoses in children and adolescents include anxiety disorders (AD), major depressive disorder (MDD), post-traumatic stress disorder (PTSD), attention-deficit/hyperactivity disorder (ADHD), and obsessive-compulsive disorder (OCD) (1). In these disorders, psychosocial interventions are recommended as first-line treatments for mild clinical presentations, whereas pharmacological therapies are generally indicated for moderate to severe cases. Within this therapeutic framework, antidepressants play a central role in the management of a subset of child and adolescent psychiatric disorders (1–4). Beyond AD, MDD, and OCD, antidepressants are also utilized for a range of additional indications, including PTSD, nocturnal enuresis, and smoking cessation. Off-label prescribing is relatively common in clinical practice; for example, amitriptyline has been used in the management of neuropathic pain, while imipramine, a tricyclic antidepressant (TCA), has historically been prescribed for ADHD (3, 5). Global antidepressant prescription rates have shown a steady upward trend, and antidepressant prescribing patterns differ across countries (6–10). Between 2005 and 2012, youth antidepressant use in the United States increased from 1.3% to 1.6%. During the same period, similar increases were observed in the United Kingdom (0.7% to 1.1%), Denmark (0.6% to 1.0%), the Netherlands (0.5% to 0.6%), and Germany (0.3% to 0.5%) (7). In Asia, the overall rate reached 4.7%, with South Korea at 1.1% (11). Rates in Western Europe ranged between 0.11% and 0.54% (12, 13), while those in the United States and Canada were 1.54% and 1.8%, respectively (14, 15). A recent study conducted in the UK reported that, following a 42% decline between 2000 and 2005, the incidence of patients newly prescribed an antidepressant began to rise from 2006 onwards. Specifically, between 2008 and 2018, the rate increased from 254.3 to 471.2 per 100,000 person-years (rate ratio 1.97, 95% confidence interval 1.96-1.99) (16). This upward trend accelerated after the onset of the COVID-19 pandemic, when depressive and anxiety symptoms increased among young people and antidepressant prescribing to children and, particularly, adolescent girls rose faster than pre-pandemic trends predicted (17–21).

Pharmacologically, antidepressants are classified into several categories, including selective serotonin reuptake inhibitors (SSRIs), serotonin-norepinephrine reuptake inhibitors (SNRIs), serotonin partial agonist/reuptake inhibitors (SPARIs), norepinephrine and dopamine reuptake inhibitors (NDRIs), selective norepinephrine reuptake inhibitors (NRIs), alpha-2 adrenoreceptor antagonists, serotonin antagonist/reuptake inhibitors (SARIs), monoamine oxidase inhibitors (MAOIs), and tricyclic antidepressants (TCAs). Although these drug classes generally exhibit comparable efficacy, they differ significantly in terms of safety and tolerability (22, 23). SSRIs represent approximately 70% of antidepressant prescriptions in pediatric populations (16).

Despite the widespread use of antidepressants, the U.S. Food and Drug Administration (FDA) has approved only a limited number for pediatric use. For example, among SSRIs, escitalopram is approved for MDD in individuals aged 12 years and older, and fluoxetine for those aged 8 years and older. Within the TCA class, clomipramine is approved for individuals aged 10 years and above. In addition, fluoxetine is approved for patients aged 7 years and older, fluvoxamine for those aged 8 years and older, and sertraline for children aged 6 years and older, specifically for OCD. Duloxetine, an SNRI, is approved for generalized anxiety disorder (GAD) in patients aged 7 years and older. While antidepressants are indicated for 32 conditions in adults, their approved indications in pediatric populations are limited to 12, leading to an increasing reliance on off-label use in clinical practice (5, 23). Regulatory labelling is not uniform across countries. In the European Union, most antidepressants are not licensed for depression or anxiety in minors; paediatric authorisations are largely limited to fluoxetine for depression and to selected agents for OCD (24). In Türkiye, the approved indications of a medicine are those specified in its nationally authorised Summary of Product Characteristics, which are generally aligned with European labelling, and off-label use is regulated by the Turkish Medicines and Medical Devices Agency (TİTCK) through a dedicated off-label drug use guideline (25).

Concomitant prescribing of antidepressants with other psychotropic agents in children and adolescents has shown a notable increase (16, 26). Systematic reviews report that 16–34% of youth receiving antidepressants in Western countries concurrently use at least one additional psychotropic medication (26). A study reported that the most frequently co-prescribed psychotropic medications with antidepressants were anxiolytics, hypnotics, and antipsychotics (16). Nevertheless, evidence-based guidelines for antidepressant-related polypharmacy strategies remain limited. Although the safety profiles and therapeutic efficacy of antidepressant-based polypharmacy have yet to be fully standardized, this approach has evolved into an indispensable strategy in contemporary clinical practice. Particularly in refractory cases and multifaceted comorbid conditions such as treatment-resistant depression, psychotic disorders, and OCD, the insufficiency of monotherapy renders combination regimens a clinical necessity (16, 27).

To date, no clinical or community-based studies in Türkiye have systematically examined antidepressant prescribing patterns, frequency of use, or polypharmacy practices among children and adolescents. This study therefore aimed to provide real-world data from a paediatric psychiatry outpatient setting in Türkiye. Accordingly, this study aims to characterize antidepressant prescribing practices, including the use of monotherapy and combination treatments, as well as associated comorbid psychiatric conditions, adverse effects, augmentation strategies, and clinical outcomes. In addition, we explored the clinical and demographic factors associated with the last recorded Clinical Global Impression (CGI) improvement rating, off-label antidepressant use and the occurrence of adverse effects within a child and adolescent psychiatry outpatient population at a Mental Health Hospital.

## Methods

### Study design, setting and participants

This single-centre, retrospective, cross-sectional chart review was reported in accordance with the STROBE statement (28). Study participants were identified from children and adolescents who had been prescribed antidepressants at the Child and Adolescent Psychiatry Outpatient Clinic of Adana Dr. Ekrem Tok Mental Health and Diseases Hospital, a public mental health institution serving a large metropolitan region in southern Türkiye. Selection was based on a review of medical records covering a 16-month period from August 1, 2020, to December 31, 2021. Patients were eligible if they were aged 0–17 years at the time of prescription, had been prescribed at least one antidepressant (Anatomical Therapeutic Chemical [ATC] code N06A, World Health Organization classification) during the study period, and had at least one follow-up visit with a documented CGI-I rating. Patients with missing or incomplete data on the study variables or clinical assessment scales were excluded. All eligible patients in the included sample were aged 4–17 years.

During the study period, the clinic recorded 5,719 outpatient visits by patients aged 0–17 years. Because this figure refers to visits rather than unique individuals, it was not used as a denominator for a patient-level prescribing rate. The number of records screened, the number excluded with reasons, and the final sample of 650 patients are shown in the participant flow diagram (**Figure 1**). For each patient, one record was abstracted, corresponding to the antidepressant treatment episode documented during the study period; the CGI-Severity (CGI-S) rating at treatment initiation and the CGI-I rating at the last recorded follow-up visit were used. Records sharing the same patient name were checked against hospital registration numbers and were confirmed to belong to different patients; thus, each of the 650 records represents a distinct patient.

**Figure 1:**
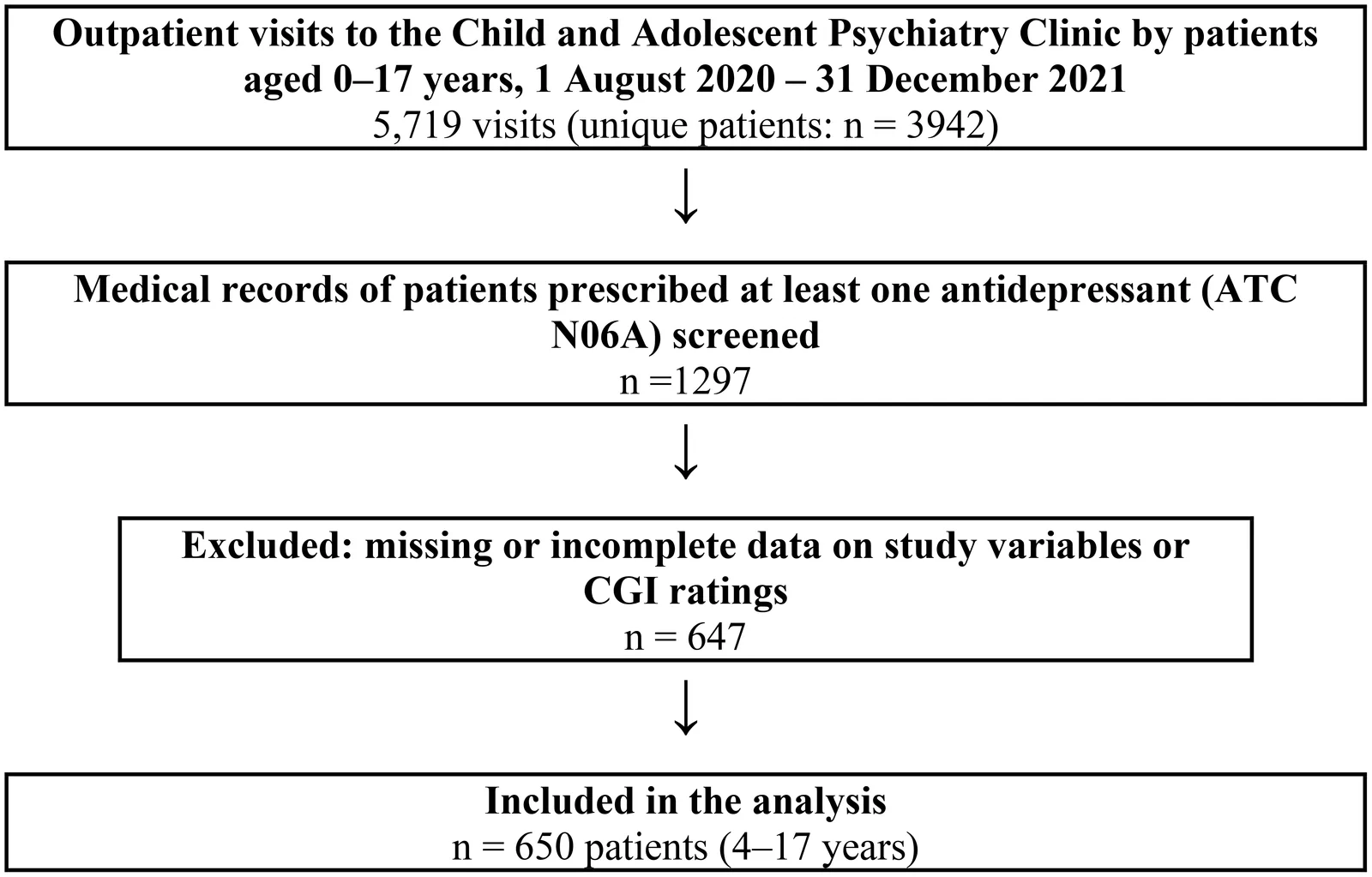
Participant flow diagram. ATC, Anatomical Therapeutic Chemical classification; CGI, Clinical Global Impression.

All data were obtained from the patients’ medical records, including demographic and clinical information as well as CGI scale scores. Ethical approval for the study was obtained from the Adana City Hospital Ethics Committee (approval date: 08.06.2023/2638).

### Clinical assessment procedures

In the public child and adolescent psychiatry outpatient clinics of Türkiye, each patient is usually followed by a single treating psychiatrist, who establishes the diagnosis by clinical interview with the child and caregivers, initiates and adjusts pharmacological treatment, and documents the clinical course at follow-up visits; structured diagnostic interviews and independent outcome raters are not part of routine care. In the present study, all patients were assessed and treated by the same child and adolescent psychiatrist (A.A.G.), who was the treating physician for the entire cohort. Diagnoses were established through unstructured clinical interviews with the child and caregivers according to the Diagnostic and Statistical Manual of Mental Disorders, Fifth Edition (DSM-5). At each visit, the clinician recorded sociodemographic variables, psychiatric diagnoses, comorbid psychiatric and medical conditions, prescribed antidepressants and dosages, concomitant medications, reported adverse effects and CGI ratings. The CGI-S was rated when antidepressant treatment was initiated, and the CGI-I was rated at follow-up visits relative to that baseline. Because the data were abstracted retrospectively, the interval between treatment initiation and the last CGI-I rating was not systematically recorded and therefore could not be quantified. Data on concurrent psychosocial interventions, including structured psychotherapy, were not systematically documented in the records and could not be analysed.

### Definitions

Off-label status was determined at the patient level for the antidepressant regimen in use at the last recorded visit, according to FDA paediatric labelling (drug, indication and age); FDA labelling was chosen as a common international reference to allow comparison with previous pharmacoepidemiological studies. A prior antidepressant trial was defined as documented discontinuation of an earlier antidepressant followed by a switch to another agent before the index episode; in 84 of the 88 patients concerned, the discontinued agent was an SSRI. Concomitant treatment was defined, as coded by the treating clinician, as the addition of another psychotropic agent to the antidepressant because of a comorbid psychiatric condition or partial response (augmentation). Individual co-prescribed psychotropic agents recorded in the charts are reported separately for all patients. Adverse effects were recorded as documented by the clinician, together with the agent to which they were attributed (the antidepressant or the co-prescribed agent).

### Measures

The CGI is a clinician-rated instrument designed to assess both the severity of illness and the overall therapeutic response on a seven-point scale (29). In the CGI-S subscale, lower scores indicate less severe illness, while a score of seven reflects an extremely ill patient. The CGI-I subscale measures treatment effectiveness, where a score of one denotes “very much improved,” and a score of seven indicates “very much worse”. In this study, CGI-S scores of 3, 4, and 5 were categorised as mildly, moderately and markedly ill, respectively. Regarding treatment response, CGI-I scores of 1 or 2 were interpreted as a positive response and a score of 3 (minimally improved) as a partial response; no patient had a CGI-I score of 4 or higher at the last recorded follow-up (29).

### Statistical analysis

Analyses were conducted using IBM SPSS Statistics for Windows, version 22.0 (IBM Corp., Armonk, NY, USA), and were verified using Python (statsmodels). Descriptive statistics are presented as mean ± standard deviation (SD), median (interquartile range) or n (%), with the denominator stated for each percentage. Categorical variables were compared using Pearson’s chi-square test or Fisher’s exact test when expected cell counts were below five, and continuous variables using the independent-samples t-test or Mann–Whitney U test, according to the distribution. Prescribing patterns were compared by sex. Three binary outcomes were examined: a CGI-I score of 1–2 at the last follow-up, off-label prescribing, and the occurrence of any adverse effect. Candidate variables were first examined in univariable logistic regression, and variables with p < 0.10 were entered into multivariable models. Concomitant treatment was not entered into the CGI-I model because concomitant agents were frequently added in response to partial improvement and would therefore represent a consequence rather than a determinant of the outcome. Age was analysed in three groups (4–10, 11–14 and 15–17 years), and the overall contribution of age group was assessed using a likelihood-ratio test. Multicollinearity was assessed using variance inflation factors (all < 1.4). Model calibration was examined with the Hosmer–Lemeshow test and discrimination with the area under the receiver operating characteristic curve (AUC). Adjusted odds ratios (aORs) with 95% confidence intervals (CIs) are reported. Because of the observational design, all regression analyses were considered exploratory, and the associations were not interpreted causally. A two-tailed p < 0.05 was considered statistically significant.

## Results

### Sample characteristics

Of the 650 patients included (**Figure 1**), 383 (58.9%) were female and 267 (41.1%) were male. The mean age was 13.23 ± 3.18 years (range 4–17); female patients were older than male patients (13.64 ± 3.01 vs. 12.65 ± 3.31 years, p < 0.001). In total, 135 patients (20.8%) were aged 4–10 years, 230 (35.4%) 11–14 years and 285 (43.8%) 15–17 years (**Table 1**). At treatment initiation, 29 patients (4.5%) were mildly, 424 (65.2%) moderately and 197 (30.3%) markedly ill according to the CGI-S.

**Table 1:** Demographic and clinical characteristics, treatment and CGI outcomes of the patients (N = 650).

Characteristic	n (%)
Age, years, mean ± SD	13.23 ± 3.18
Age group	
4–10 years	135 (20.8)
11–14 years	230 (35.4)
15–17 years	285 (43.8)
Sex	
Female	383 (58.9)
Male	267 (41.1)
Psychiatric comorbidity	
Yes	467 (71.8)
No	183 (28.2)
Physical comorbidity	
Yes	38 (5.8)
No	612 (94.2)
Labelling status of current antidepressant regimen (FDA)	
On-label	164 (25.2)
Off-label	486 (74.8)
Labelling status of first antidepressant (FDA)	
On-label	179 (27.5)
Off-label	471 (72.5)
Labelling status of second antidepressant (FDA), n = 87	
On-label	19 (21.8)
Off-label	68 (78.2)
CGI-Severity at treatment initiation	
Mildly ill (3)	29 (4.5)
Moderately ill (4)	424 (65.2)
Markedly ill (5)	197 (30.3)
CGI-Improvement at last recorded follow-up	
Positive response (score 1–2)	550 (84.6)
Very much improved (1)	311 (47.8)
Much improved (2)	239 (36.8)
Partial response: minimally improved (3)	100 (15.4)
Concomitant psychotropic treatment	
No (monotherapy)	365 (56.2)
Yes	285 (43.8)
Any adverse effect	
Yes	139 (21.4)
No	511 (78.6)
Prior antidepressant trial	
Yes	88 (13.5)
No	562 (86.5)
Reason for discontinuation of prior antidepressant, n = 88	
Lack of efficacy	63 (71.6)
Partial efficacy	10 (11.4)
Adverse effects	15 (17.0)

Percentages are calculated with N = 650 as the denominator unless another denominator (n) is stated. CGI, Clinical Global Impression; FDA, U.S. Food and Drug Administration; SD, standard deviation. Labelling status was classified according to FDA paediatric labelling (drug, indication and age). Prior antidepressant trial: discontinuation of an earlier antidepressant followed by a switch to another agent (the discontinued agent was an SSRI in 84 of 88 patients).

At least one anxiety disorder was diagnosed in 272 patients (41.8%), most commonly GAD (n = 171, 26.3%); MDD was diagnosed in 246 (37.8%) and ADHD in 187 (28.8%) (**Table 2**). Psychiatric comorbidity was present in 467 patients (71.8%) and physical comorbidity in 38 (5.8%) (**Table 1**).

**Table 2:** Psychiatric diagnoses of the patients (N = 650).

Diagnosis*	n (%)
Any anxiety disorder†	272 (41.8)
Generalised anxiety disorder	171 (26.3)
Social anxiety disorder	59 (9.1)
Test anxiety	42 (6.5)
Specific phobia	34 (5.2)
Separation anxiety disorder	13 (2.0)
School refusal (school phobia)	9 (1.4)
Panic disorder	8 (1.2)
Major depressive disorder	246 (37.8)
Attention-deficit/hyperactivity disorder	187 (28.8)
Conduct disorder	121 (18.6)
Obsessive-compulsive disorder	87 (13.4)
Intellectual disability/borderline intellectual functioning‡	58 (8.9)
Enuresis nocturna	42 (6.5)
Adjustment disorder	36 (5.5)
Sleep disorders	34 (5.2)
Post-traumatic stress disorder	32 (4.9)
Oppositional defiant disorder	23 (3.5)
Stuttering	21 (3.2)
Conversion disorder	17 (2.6)
Personality organisation§	13 (2.0)
Masturbation behaviour	12 (1.8)
Encopresis	9 (1.4)
Autism spectrum disorder	9 (1.4)
Tic disorders	9 (1.4)
Trichotillomania	7 (1.1)
Specific learning disorder	6 (0.9)
Bipolar disorder	5 (0.8)
Disruptive mood dysregulation disorder	5 (0.8)
Anorexia nervosa	4 (0.6)
Somatic symptom disorder	4 (0.6)
Acute stress disorder	1 (0.2)
Other conditions¶	38 (5.8)

*Patients could have more than one diagnosis; therefore, the number of diagnoses exceeds the number of patients. Percentages are calculated with N = 650 as the denominator. †Number of patients with at least one anxiety disorder; subtypes are not mutually exclusive. 272 patients had a total of 336 anxiety diagnosis (1diagnosis: n=216; 2: n=48; 3: n=8). ‡Mild (n = 34), moderate (n = 15) and severe (n = 2) intellectual disability and borderline intellectual functioning (n = 7). §Borderline (n = 11) and obsessive-compulsive (n = 2) personality organisation. ¶Dissociative symptoms (n = 23), selective mutism (n = 8), substance use disorder (n = 5), gender dysphoria (n = 1) and articulation disorder (n = 1).

### Antidepressant prescribing, off-label use and sex differences

In total, 676 antidepressant prescriptions were recorded for the 650 patients (27 patients received two agents concurrently). At least one SSRI was prescribed to 601 patients (92.5%), and SSRIs accounted for 88.9% of all antidepressant prescriptions. The most commonly used agents were fluoxetine (n = 313, 48.2%) and sertraline (n = 222, 34.2%) (**Table 3**). Prescribing was off-label according to FDA labelling in 486 patients (74.8%) and on-label in 164 (25.2%) (**Table 1**).

**Table 3:** Antidepressants prescribed, doses and distribution by sex.

Antidepressant	Total, n (%) N = 650	Dose, mg/day, mean ± SD	Dose range, mg/day	Female, n (%) n = 383	Male, n (%) n = 267	p†
Fluoxetine *	313 (48.2)	22.59 ± 9.99	5–60	179 (46.7)	134 (50.2)	0.386
Sertraline *	222 (34.2)	67.07 ± 32.98	25–200	145 (37.9)	77 (28.8)	0.017
Mirtazapine	44 (6.8)	19.77 ± 10.62	7.5–45	17 (4.4)	27 (10.1)	0.005
Escitalopram *	41 (6.3)	12.68 ± 7.01	2.5–40	32 (8.4)	9 (3.4)	0.010
Imipramine	21 (3.2)	25.60 ± 6.22	12.5–50	11 (2.9)	10 (3.7)	0.536
Paroxetine *	16 (2.5)	28.12 ± 15.59	10–60	5 (1.3)	11 (4.1)	0.023
Citalopram *	7 (1.1)	21.43 ± 3.78	20–30	2 (0.5)	5 (1.9)	0.130
Venlafaxine	6 (0.9)	187.50 ± 91.86	75–300	4 (1.0)	2 (0.7)	1.000
Duloxetine	3 (0.5)	50.00 ± 17.32	30–60	3 (0.8)	0 (0.0)	0.273
Fluvoxamine *	2 (0.3)	150.00 ± 70.71	100–200	0 (0.0)	2 (0.7)	0.168
Trazodone	1 (0.2)	50.00	–	0 (0.0)	1 (0.4)	0.411
Any SSRI	601 (92.5)			363 (94.8)	238 (89.1)	0.007

*SSRI. In total, 676 antidepressant prescriptions were recorded for 650 patients because 27 patients received two antidepressants concurrently; therefore, the column totals exceed the number of patients. †Pearson chi-square test, or Fisher’s exact test when an expected cell count was < 5. SD, standard deviation; SSRI, selective serotonin reuptake inhibitor.

Sertraline (37.9% vs. 28.8%, p = 0.017) and escitalopram (8.4% vs. 3.4%, p = 0.010) were prescribed more often to female patients, whereas mirtazapine (10.1% vs. 4.4%, p = 0.005) and paroxetine (4.1% vs. 1.3%, p = 0.023) were prescribed more often to male patients; fluoxetine use did not differ by sex (46.7% vs. 50.2%, p = 0.386). Overall, at least one SSRI was prescribed to 94.8% of female and 89.1% of male patients (p = 0.007) (**Table 3**). Off-label prescribing (72.8% vs. 77.5%, p = 0.176) and concomitant treatment (44.9% vs. 42.3%, p = 0.513) did not differ by sex. Among co-prescribed drug classes, ADHD medications were recorded more often in male patients (31.5% vs. 16.4%, p < 0.001) and mood stabilisers in female patients (3.7% vs. 1.1%, p = 0.047), whereas antipsychotic use was similar (34.5% vs. 39.0%, p = 0.242).

### Concomitant psychotropic treatment

Antidepressant monotherapy was recorded in 365 patients (56.2%), and 285 (43.8%) received concomitant psychotropic treatment. Among the latter, the reasons were a comorbid psychiatric condition (n = 158, 55.4%), partial response (n = 125, 43.9%) or not specified (n = 2, 0.7%). Among the 285 patients receiving concomitant treatment, the most frequently added agents were risperidone (38.2%), aripiprazole (20.7%), OROS methylphenidate (11.6%) and quetiapine (10.2%). Across all 650 patients, at least one other psychotropic agent was recorded in 349 (53.7%), mainly antipsychotics (36.3%) and ADHD medications (22.6%) (**Table 4**).

**Table 4:** Concomitant psychotropic treatment, co-prescribed agents and CGI-I outcome by treatment type.

Variable	All patients, n (%) N = 650	Concomitant treatment, n (%) n = 285
Concomitant treatment (clinician-coded)		
No (antidepressant monotherapy)	365 (56.2)	–
Yes	285 (43.8)	–
Reason for concomitant treatment, n = 285		
Comorbid psychiatric condition	–	158 (55.4)
Partial response (augmentation)	–	125 (43.9)
Not specified	–	2 (0.7)
Co-prescribed psychotropic agents recorded		
Any co-prescribed psychotropic agent	349 (53.7)	266 (93.3)
**Antipsychotics**	**236 (36.3)**	**229 (80.4)**
Risperidone	114 (17.5)	109 (38.2)
Aripiprazole	61 (9.4)	59 (20.7)
Quetiapine	29 (4.5)	29 (10.2)
Haloperidol	19 (2.9)	19 (6.7)
Olanzapine	15 (2.3)	15 (5.3)
Zuclopenthixol	1 (0.2)	1 (0.4)
Chlorpromazine	1 (0.2)	1 (0.4)
ADHD medications	147 (22.6)	67 (23.5)
OROS methylphenidate	64 (9.8)	33 (11.6)
Immediate-release methylphenidate	34 (5.2)	6 (2.1)
Atomoxetine	49 (7.5)	28 (9.8)
Mood stabilisers	17 (2.6)	17 (6.0)
Sodium valproate	11 (1.7)	11 (3.9)
Lithium	4 (0.6)	4 (1.4)
Carbamazepine	2 (0.3)	2 (0.7)
Benzodiazepines	15 (2.3)	15 (5.3)
Alprazolam	8 (1.2)	8 (2.8)
Clonazepam	5 (0.8)	5 (1.8)
Lorazepam	1 (0.2)	1 (0.4)
Diazepam	1 (0.2)	1 (0.4)
Other agents	24 (3.7)	24 (8.4)
Propranolol	24 (3.7)	24 (8.4)
Hydroxyzine	1 (0.2)	1 (0.4)
CGI-I at last recorded follow-up		
Positive response (1–2), monotherapy, n = 365	363 (99.5)	–
Positive response (1–2), concomitant treatment, n = 285	–	187 (65.6)

Concomitant treatment was coded by the treating clinician as the addition of another psychotropic agent because of a comorbid psychiatric condition or partial response. Co-prescribed agents are those recorded in the medical record at the time of antidepressant treatment; some patients coded as receiving monotherapy had an ADHD medication (or, rarely, an antipsychotic) recorded, and some patients received more than one co-prescribed agent. CGI-I, Clinical Global Impression–Improvement; OROS, osmotic-release oral system.

### Adverse effects

Adverse effects were recorded in 139 patients (21.4%): in 43 of 365 patients (11.8%) receiving monotherapy and in 96 of 285 (33.7%) receiving concomitant treatment (p < 0.001). The clinician attributed the adverse effect to the antidepressant in 48 patients and to the co-prescribed agent in 91. No adverse effect was rated as outweighing the therapeutic benefit; 18 patients, all with adverse effects attributed to a co-prescribed agent, had adverse effects rated as significantly interfering with functioning. Sedation (n = 76) and weight gain (n = 63) were the most frequent adverse effects and were more common with concomitant treatment (both p < 0.001), whereas bruxism occurred almost exclusively with monotherapy (8 of 9 cases) (**Table 5**). By agent, adverse effects were recorded in 17.9% of patients receiving fluoxetine, 20.7% receiving sertraline, 14.6% receiving escitalopram, 56.8% receiving mirtazapine and 50.0% receiving paroxetine; among patients receiving monotherapy, the corresponding rates were 12.2% for fluoxetine and 4.0% for sertraline (**Table 6**). Sedation and weight gain predominated with mirtazapine, whereas bruxism was observed only with fluoxetine. No suicidal ideation or suicide attempts were documented.

**Table 5:** Adverse effects according to treatment type.

Adverse effect	Monotherapy, n (%) n = 365	Concomitant treatment, n (%) n = 285	Total, n (%) N = 650	p*
Any adverse effect	43 (11.8)	96 (33.7)	139 (21.4)	<0.001
Sedation	14 (3.8)	62 (21.8)	76 (11.7)	<0.001
Weight gain	9 (2.5)	54 (18.9)	63 (9.7)	<0.001
Loss of appetite	9 (2.5)	3 (1.1)	12 (1.8)	0.184
Bruxism	8 (2.2)	1 (0.4)	9 (1.4)	0.085
Palpitations	5 (1.4)	2 (0.7)	7 (1.1)	0.475
Nausea	2 (0.5)	2 (0.7)	4 (0.6)	1.000
Sexual dysfunction	0 (0.0)	3 (1.1)	3 (0.5)	0.084
Akathisia	1 (0.3)	1 (0.4)	2 (0.3)	1.000
Hyperprolactinaemia	0 (0.0)	2 (0.7)	2 (0.3)	0.192
Headache	1 (0.3)	0 (0.0)	1 (0.2)	1.000
QTc prolongation	0 (0.0)	1 (0.4)	1 (0.2)	0.438
Vomiting	1 (0.3)	0 (0.0)	1 (0.2)	1.000
Dry mouth	1 (0.3)	0 (0.0)	1 (0.2)	1.000
Other	0 (0.0)	3 (1.1)	3 (0.5)	0.084

Adverse effects were counted among the 139 patients with a recorded adverse effect; a patient could have more than one adverse effect. Percentages use the group size shown in the column heading as denominator. *Pearson chi-square test, or Fisher’s exact test when an expected cell count was < 5.

**Table 6:** Adverse effects by antidepressant agent.

Antidepressant	Patients, n	Any adverse effect, all patients, n/N (%)	Any adverse effect, monotherapy, n/N (%)	Any adverse effect, concomitant treatment, n/N (%)	Most frequent adverse effects, n
Fluoxetine	313	56/313 (17.9)	26/213 (12.2)	30/100 (30.0)	Sedation (23), Weight gain (15), Bruxism (9)
Sertraline	222	46/222 (20.7)	4/101 (4.0)	42/121 (34.7)	Sedation (29), Weight gain (25), Loss of appetite (4)
Mirtazapine	44	25/44 (56.8)	9/14 (64.3)	16/30 (53.3)	Weight gain (22), Sedation (15), Loss of appetite (1)
Escitalopram	41	6/41 (14.6)	1/13 (7.7)	5/28 (17.9)	Sedation (3), Weight gain (3), Akathisia (1)
Imipramine	21	5/21 (23.8)	5/18 (27.8)	0/3 (0.0)	Sedation (4), Palpitations (1), Dry mouth (1)
Paroxetine	16	8/16 (50.0)	1/4 (25.0)	7/12 (58.3)	Weight gain (6), Sedation (4), Sexual dysfunction (1)
Citalopram	7	1/7 (14.3)	0/4 (0.0)	1/3 (33.3)	Sedation (1)
Venlafaxine	6	2/6 (33.3)	–	2/6 (33.3)	Sedation (2), Weight gain (1)
Duloxetine	3	0/3 (0.0)	0/2 (0.0)	0/1 (0.0)	–
Fluvoxamine	2	1/2 (50.0)	–	1/2 (50.0)	Sedation (1), Weight gain (1)
Trazodone	1	1/1 (100.0)	–	1/1 (100.0)	Weight gain (1)

Each row includes all patients who received the agent (27 patients received two antidepressants and are counted under both agents). Adverse effects occurring during concomitant treatment may be attributable to the co-prescribed agent. Descriptive data; no statistical comparison was made between agents because of small numbers.

### Clinical outcome and prior antidepressant trials

At the last recorded follow-up, 311 patients (47.8%) were rated as very much improved, 239 (36.8%) as much improved and 100 (15.4%) as minimally improved on the CGI-I; thus, 550 patients (84.6%) had a CGI-I score of 1–2 (**Table 1**). A CGI-I score of 1–2 was recorded in 363 of 365 patients (99.5%) receiving monotherapy and in 187 of 285 (65.6%) receiving concomitant treatment; 98 of the 100 patients with a CGI-I score of 3 were receiving concomitant treatment.

A prior antidepressant trial was documented in 88 patients (13.5%). The reasons for discontinuation were lack of efficacy (n = 63, 71.6%), partial efficacy (n = 10, 11.4%) and adverse effects (n = 15, 17.0%). The discontinued agents were most commonly fluoxetine (n = 47, 53.4%), sertraline (n = 25, 28.4%) and escitalopram (n = 5, 5.7%), and patients were most often switched to sertraline (n = 28, 31.8%), escitalopram (n = 21, 23.9%), paroxetine (n = 15, 17.0%) or fluoxetine (n = 14, 15.9%).

### Factors associated with CGI-I response, off-label prescribing and adverse effects

In the multivariable model for a CGI-I score of 1–2 (**Table 7**), patients aged 11–14 years (aOR 0.37, 95% CI 0.17–0.83; p = 0.016) and 15–17 years (aOR 0.28, 95% CI 0.13–0.61; p = 0.001) had lower odds than those aged 4–10 years (likelihood-ratio test for age group, p = 0.002), and a prior antidepressant trial was associated with lower odds (aOR 0.46, 95% CI 0.27–0.78; p = 0.004). Sex, off-label use, adverse effects, baseline CGI-S and diagnoses were not associated with the outcome in univariable analyses. The estimates were similar after additional adjustment for baseline CGI-S (age 11–14 years: aOR 0.34; 15–17 years: aOR 0.25; prior antidepressant trial: aOR 0.42). The model showed acceptable calibration (Hosmer–Lemeshow p = 0.608) but limited discrimination (AUC 0.64).

**Table 7:** Univariable and multivariable logistic regression analyses of factors associated with clinical outcomes.

Variable	Univariable OR (95% CI)	p	Multivariable aOR (95% CI)	p
Outcome 1: CGI-I score of 1–2 at last recorded follow-up (550/650)				
Age 4–10 years	Reference		Reference	
Age 11–14 years	0.36 (0.16–0.81)	0.013	0.37 (0.17–0.83)	0.016
Age 15–17 years	0.25 (0.11–0.53)	<0.001	0.28 (0.13–0.61)	0.001
Female sex	0.86 (0.55–1.33)	0.497	–	
Prior antidepressant trial	0.39 (0.23–0.65)	<0.001	0.46 (0.27–0.78)	0.004
Off-label prescribing	0.99 (0.60–1.61)	0.954	–	
Any adverse effect	1.03 (0.61–1.73)	0.919	–	
CGI-S moderately ill (vs mildly)	1.32 (0.52–3.36)	0.561	–	
CGI-S markedly ill (vs mildly)	1.88 (0.70–5.08)	0.213	–	
Major depressive disorder	0.94 (0.61–1.46)	0.796	–	
Generalised anxiety disorder	1.23 (0.75–2.04)	0.415	–	
ADHD	0.75 (0.48–1.18)	0.210	–	
Psychiatric comorbidity	0.68 (0.41–1.13)	0.139	–	
Concomitant treatment‖	0.01 (0.00–0.04)	<0.001	–	
Outcome 2: Off-label prescribing (486/650)				
Age 4–10 years	Reference		–	
Age 11–14 years	0.72 (0.43–1.21)	0.220	–	
Age 15–17 years	0.66 (0.40–1.09)	0.105	–	
Female sex	0.78 (0.54–1.12)	0.177	–	
Prior antidepressant trial	1.17 (0.69–2.00)	0.561	–	
CGI-I score of 1–2	0.99 (0.60–1.61)	0.954	–	
Psychiatric comorbidity	0.99 (0.67–1.47)	0.972	–	
ADHD	1.68 (1.10–2.55)	0.016	1.46 (0.94–2.28)	0.096
Generalised anxiety disorder	4.01 (2.34–6.86)	<0.001	2.41 (1.35–4.30)	0.003
Major depressive disorder	0.23 (0.16–0.33)	<0.001	0.31 (0.21–0.47)	<0.001
Outcome 3: Any adverse effect (139/650)				
Age 4–10 years	Reference		Reference	
Age 11–14 years	1.25 (0.72–2.17)	0.427	0.90 (0.50–1.62)	0.728
Age 15–17 years	1.56 (0.92–2.63)	0.099	0.87 (0.48–1.57)	0.644
Female sex	0.77 (0.53–1.13)	0.180	–	
Prior antidepressant trial	2.29 (1.41–3.71)	<0.001	1.69 (1.00–2.86)	0.051
Concomitant treatment	3.80 (2.54–5.69)	<0.001	3.32 (2.14–5.16)	<0.001
Major depressive disorder	1.14 (0.78–1.67)	0.503	–	
Generalised anxiety disorder	0.49 (0.30–0.79)	0.004	0.58 (0.35–0.97)	0.038
ADHD	1.96 (1.32–2.90)	<0.001	1.77 (1.17–2.69)	0.007
Off-label prescribing	1.16 (0.75–1.81)	0.499	–	

Variables with p < 0.10 in univariable analysis were entered into the multivariable models. Outcome 1 model: age group and prior antidepressant trial (likelihood-ratio test for age group, p = 0.002; Hosmer–Lemeshow p = 0.608; AUC 0.64). Outcome 2 model: ADHD, GAD and MDD (Hosmer–Lemeshow p = 0.523; AUC 0.71). Outcome 3 model: age group, prior antidepressant trial, concomitant treatment, GAD and ADHD (Hosmer–Lemeshow p = 0.005; AUC 0.71). ‖Not entered into the multivariable model because concomitant agents were frequently added in response to partial improvement (reverse causation). Associations are exploratory and should not be interpreted causally. ADHD, attention-deficit/hyperactivity disorder; aOR, adjusted odds ratio; AUC, area under the receiver operating characteristic curve; CGI-I/S, Clinical Global Impression–Improvement/Severity; CI, confidence interval; GAD, generalised anxiety disorder; MDD, major depressive disorder; OR, odds ratio. p values < 0.05 are statistically significant.

In the multivariable model for off-label prescribing, GAD was associated with higher odds (aOR 2.41, 95% CI 1.35–4.30; p = 0.003) and MDD with lower odds (aOR 0.31, 95% CI 0.21–0.47; p < 0.001) of off-label prescribing, whereas the association with ADHD was attenuated and not statistically significant (aOR 1.46, 95% CI 0.94–2.28; p = 0.096). Psychiatric comorbidity was not associated with off-label prescribing (OR 0.99, p = 0.972) (**Table 7**).

In the multivariable model for adverse effects, concomitant treatment (aOR 3.32, 95% CI 2.14–5.16; p < 0.001) and ADHD (aOR 1.77, 95% CI 1.17–2.69; p = 0.007) were associated with higher odds, and GAD with lower odds (aOR 0.58, 95% CI 0.35–0.97; p = 0.038), of adverse effects. The association of a prior antidepressant trial with adverse effects, which was significant in univariable analysis (OR 2.29, 95% CI 1.41–3.71), was attenuated after adjustment (aOR 1.69, 95% CI 1.00–2.86; p = 0.051). Age group was not associated with adverse effects (**Table 7**). This model showed adequate discrimination (AUC 0.71) but suboptimal calibration (Hosmer–Lemeshow p = 0.005).

## Discussion

To our knowledge, this is the first study to describe antidepressant prescribing among children and adolescents treated at a child and adolescent psychiatry outpatient clinic of a mental health hospital in Türkiye. Beyond describing prescribing patterns, we evaluated the clinical and demographic factors associated with treatment response (CGI-I), off-label antidepressant prescribing, and the occurrence of adverse effects. These findings provide novel insights into real-world prescribing practices and contribute to the limited evidence on pediatric antidepressant use in Türkiye.

In the present study, prescribing was concentrated among adolescents in the 15–17 age range and female patients, was dominated by SSRIs, was off-label in three-quarters of patients and was combined with other psychotropic agents in more than two-fifths. Adverse effects were recorded in about one in five patients and were strongly linked to concomitant treatment. Younger age was associated with a more favourable last recorded CGI-I rating. These findings describe a single clinical setting and should be interpreted as exploratory associations rather than as estimates of treatment effects.

The predominance of adolescents and female patients is consistent with population-based data showing the highest antidepressant use among adolescent girls (16). This pattern may have been accentuated by the timing of our study: the rise in antidepressant dispensing after March 2020 was largely driven by adolescent girls, whereas dispensing to adolescent boys remained stable or declined (19). Antidepressants were used for a variety of psychiatric conditions in the present cohort, most commonly for AD, depression, and ADHD, which is consistent with existing literature (3, 5, 9). Among AD, prescriptions were most frequent for GAD. The continued preference for SSRIs in these indications aligns with their well-established efficacy and relatively favorable tolerability, supporting their position as first-line treatment options in current clinical guidelines (2, 4).

The predominance of SSRIs, particularly fluoxetine and sertraline, also mirrors international data and reflects the evidence base and tolerability of these agents in youth (3, 5, 9, 30, 31). The clinical preference for SSRIs is supported by their pharmacokinetic characteristics, effectiveness at low doses, minimal dose-dependent adverse effect escalation, and low toxicity risk (3, 23). Their increased use in children and adolescents also stems from the limited range of pharmacological options approved for this population and the robust evidence base supporting SSRIs in randomized controlled trials. Notably, fluoxetine was the first SSRI approved by the U.S. FDA for the treatment of MDD in pediatric patients, a milestone that has had a substantial impact on antidepressant prescribing practices. The relatively mild nature of SSRI-associated adverse effects -such as behavioral activation, appetite changes, or gastrointestinal symptoms- further supports their widespread use in youth populations (23, 30). The sex differences in individual agents should be interpreted in the light of the older age and diagnostic profile of female patients; for example, the more frequent use of mirtazapine and paroxetine in boys and of escitalopram in girls may reflect differences in comorbidity (such as ADHD, sleep problems or conduct problems) rather than sex per se. The much higher use of ADHD medications in boys is consistent with the sex distribution of ADHD.

Three-quarters of the patients received an off-label regimen according to FDA labelling, a proportion comparable to that reported in European primary care (13). Because paediatric antidepressant labelling in Europe and Türkiye is generally narrower than in the United States—for example, escitalopram and duloxetine have paediatric indications in the United States but not in Europe (24)—the off-label proportion would probably have been higher if national labelling had been applied. In the multivariable model, GAD was associated with higher and MDD with lower odds of off-label prescribing. This largely reflects the regulatory framework itself: fluoxetine and escitalopram are labelled for paediatric MDD, whereas no SSRI is labelled for non-OCD anxiety disorders in youth despite supportive trial evidence and guideline recommendations (2, 30). The initially observed association with ADHD was attenuated after adjustment for mood and anxiety diagnoses, suggesting that it was largely explained by co-occurring internalising disorders, for which antidepressants are commonly added in youth with ADHD (32–34). Accordingly, off-label prescribing in this sample appears to reflect the gap between regulatory approvals and the clinical needs of comorbid patients rather than inappropriate prescribing, although indication-level appropriateness could not be assessed.

In the current study, concomitant psychotropic treatment was common and involved mainly antipsychotics and ADHD medications. In clinical practice, combination strategies involving antidepressants and other psychotropic agents are increasingly employed for complex or treatment-resistant cases. Such regimens may be indicated in the presence of psychiatric comorbidities or in more severe clinical presentations, including treatment-resistant depression, OCD, or psychotic depression. However, evidence supporting the safety and efficacy of polypharmacy in this population remains limited, with most available data derived from open-label trials and case series (27). In the study cohort, the frequent co-prescription of risperidone and aripiprazole is consistent with reports of increasing antidepressant–antipsychotic co-prescribing in adolescents, often for irritability, aggression or self-injurious behaviour, despite limited supporting evidence (26, 35–41). In addition, medications for ADHD represented the second most commonly co-prescribed drug class after antipsychotics, which is likely attributable to the frequent co-occurrence of ADHD with depression, AD, and OCD. Consistent with the findings of the present study, previous studies have reported that psychostimulants are frequently co-prescribed with antidepressants (11, 26).

Adverse effects were almost three times as frequent with concomitant treatment as with monotherapy, and concomitant treatment remained the strongest correlate of adverse effects after adjustment. Sedation and weight gain—effects typical of antipsychotics and of mirtazapine rather than of SSRIs—accounted for most adverse effects, and the clinician attributed two-thirds of all adverse effects to the co-prescribed agent. In contrast, the adverse effect rates of fluoxetine and sertraline monotherapy were low, and bruxism, a recognised SSRI-related effect, occurred almost exclusively with fluoxetine monotherapy (42–44). These findings support careful monitoring of metabolic and sedative effects when antidepressants are combined with antipsychotics (26, 42). The finding that ADHD was independently associated with adverse effects may reflect co-prescription of stimulants, which can cause appetite loss and other adverse effects, as well as greater clinical complexity. No suicidal ideation or suicide attempts were documented. This finding may be associated with several factors, including the outpatient status of the patient population, the presence of regular and careful psychiatric follow-up, the heterogeneity of diagnostic groups and symptom severity, family support and parental supervision, underreporting, and sociocultural characteristics. However, given the retrospective design and the possibility of under-recording, this finding does not permit conclusions about suicidality risk, and monitoring after antidepressant initiation remains essential (45–48).

A CGI-I score of 1–2 was recorded in 84.6% of patients at the last recorded follow-up. This proportion should not be interpreted as an estimate of antidepressant efficacy. The study had no control group, and placebo response rates in paediatric antidepressant trials are substantial (30, 48); 43.8% of patients received concomitant psychotropic medications; the interval between treatment initiation and the last CGI-I rating was not standardised; the information on psychosocial interventions was incomplete; and ratings were made by the unblinded treating clinician. Furthermore, because patients who did not improve sufficiently were usually given additional medication—98 of the 100 patients with a CGI-I score of 3 were receiving concomitant treatment—the CGI-I rating and treatment intensity are closely intertwined. The figure is best regarded as a descriptive summary of the clinical course documented in routine practice.

Younger age was associated with higher odds of a CGI-I score of 1–2, and this association was robust to adjustment for baseline severity. Several explanations are possible, including differences in diagnostic composition, greater parental supervision of medication adherence in younger children, and the more complex symptom patterns, higher comorbidity, longer illness duration and greater psychosocial stress of adolescence (49–52). This finding contrasts with meta-analytic evidence, which suggests more consistent drug–placebo differences in adolescents than in children, partly because of higher placebo response in children, although fluoxetine has shown efficacy in both age groups (48, 53, 54). Our finding may therefore reflect differences in the natural course of illness or in non-specific treatment effects across age groups rather than differences in pharmacological response.

A prior antidepressant trial was associated with lower odds of a CGI-I score of 1–2 and, in unadjusted analyses, with higher odds of adverse effects. Previous treatment is, however, a marker of treatment history, illness complexity, severity and prior non-response or intolerance, and patients with a prior trial more often received concomitant treatment (72.7% vs. 39.3%). Consistent with this, the association with adverse effects was attenuated to borderline significance after adjustment for concomitant treatment, and the association with CGI-I response may likewise reflect unmeasured differences in illness severity, chronicity and prior non-response. These associations are therefore observational and should not be interpreted causally; they do not indicate that previous SSRI exposure itself reduces treatment response or increases vulnerability to adverse effects. The switching pattern observed, predominantly from one SSRI to another, is consistent with guideline recommendations and with the TORDIA trial for adolescents with SSRI-resistant depression (4, 55).

The study period (August 2020 to December 2021) fell entirely within the COVID-19 pandemic, an important contextual factor for interpreting our findings. Meta-analyses have documented increases in depressive and anxiety symptoms among young people during the pandemic, particularly among adolescents and girls (17, 18), and population-based studies from the United States, Denmark, France, Australia and Finland have reported accelerated increases in antidepressant and other psychotropic prescribing to youth after March 2020 (19–21, 56, 57). In Türkiye, this period also involved prolonged school closures with distance education, restrictions on the movement of minors and disruption of in-person health services, which may have limited access to psychosocial interventions and influenced the threshold for pharmacological treatment. Changes in referral patterns, symptom profiles and follow-up intensity during the pandemic may have affected the age and sex distribution, the diagnostic mix, the frequency of concomitant treatment and the outcomes observed, and pandemic-related stressors may have affected age groups differently. Because all data were collected during the pandemic and no pre-pandemic comparison period was available, we cannot determine to what extent the patterns observed reflect the pandemic rather than usual practice, and our findings may not be generalisable to non-pandemic periods.

### Strengths and limitations

The strengths of this study include a relatively large clinical sample, face-to-face psychiatric evaluation of all patients according to DSM-5 criteria, detailed information on individual agents, doses, co-prescribed drugs and adverse effects, and a uniform rating approach, as a single clinician applied the same diagnostic criteria and CGI ratings throughout. The study also provides the first clinical data on paediatric antidepressant prescribing in Türkiye.

Several limitations should be considered. First, this was a single-centre, retrospective study conducted at a public mental health hospital in southern Türkiye, and only patients who had been prescribed antidepressants and had complete clinical data were included. These features may have introduced selection bias—for example, if patients with incomplete records differed in severity, adherence or outcome—and the findings should be interpreted within the context of this specific clinical setting and patient group. Second, the study was conducted entirely during the COVID-19 pandemic, as discussed above. Third, diagnoses were based on unstructured clinical interviews rather than structured instruments such as the Kiddie Schedule for Affective Disorders and Schizophrenia for School-Age Children–Present and Lifetime Version, and all assessments were made by a single, unblinded treating clinician; inter-rater reliability could therefore not be assessed, and observer and expectation bias cannot be excluded. Fourth, only the last recorded CGI-I rating was used, and the interval between treatment initiation and this assessment varied between patients and was not quantified, which may affect the comparability of outcomes; the CGI is also a global measure that may not capture disorder-specific change. Fifth, there was no control group, and information on psychotherapy and other psychosocial interventions, adherence and treatment duration was not available. Sixth, off-label status was classified according to FDA rather than national labelling. Seventh, adverse effects were recorded as spontaneously reported and documented in routine practice, which may have led to under-ascertainment, and the adverse effect model showed suboptimal calibration.

## Conclusion

In this single-centre sample of children and adolescents treated with antidepressants in Türkiye, prescribing was more common among female patients and adolescents aged 15–17 years, was dominated by SSRIs—particularly fluoxetine and sertraline—and was off-label in approximately three-quarters of prescriptions and frequently combined with other psychotropic agents. Antidepressants appear to be generally effective and well tolerated in this population. Younger age was associated with a more favourable last recorded CGI-I rating, whereas a prior antidepressant trial was associated with a less favourable CGI-I rating, probably as a marker of more complex or treatment-resistant illness. GAD and MDD were strongly associated with off-label use. Concomitant treatment was the main correlate of adverse effects, which consisted mainly of sedation and weight gain. Because of the retrospective, uncontrolled design, these findings are descriptive and exploratory and should not be interpreted as evidence of antidepressant efficacy or causal effects. Nevertheless, this study provides insight into the clinical reality in Türkiye, where practitioners must navigate the gap between regulatory approvals and the complex, frequently comorbid presentations of pediatric patients, with SSRIs remaining a commonly used treatment option despite high rates of off-label prescribing. Prospective, multicentre studies with structured diagnostic assessment, standardised outcome measurement and follow-up intervals, and documentation of psychosocial treatment are needed to guide antidepressant prescribing in children and adolescents in Türkiye.

## Data Availability

The raw data supporting the conclusions of this article will be made available by the authors, without undue reservation.
